# A Preliminary Study of Interdisciplinary Approach with a Single-Stage Surgery in Children with Cleft Lip and Palate

**DOI:** 10.3390/jpm12101741

**Published:** 2022-10-20

**Authors:** Takashi Matsumura, Hitoshi Kawanabe, Naoko Nemoto, Saki Ogino, Kazunori Fukui, Akihiko Oyama, Toru Okamoto

**Affiliations:** 1 Department of Orthodontics and Dentofacial Orthopedics, Graduate School of Dentistry, Ohu University, 31-1 Triangular Hall, Tomita-cho, Koriyama 963-8611, Fukushima, Japan; 2 Division of Orthodontics and Dentofacial Orthopedics, Department of Oral Growth and Development, School of Dentistry, Ohu University, 31-1 Triangular Hall, Tomita-cho, Koriyama 963-8611, Fukushima, Japan; 3 Department of Plastic and Reconstructive Surgery, Fukushima Medical University, 1 Hikarigaoka, Fukushima 960-1295, Fukushima, Japan; 4 Okamoto Orthodontic Clinic, 3-25 Kita 1-jo Nishi 3-chome, Sapporo-shi 060-0001, Hokkaido, Japan

**Keywords:** cleft lip and palate, preoperative orthognathic treatment, first-stage surgery, alveolar cleft bone graft

## Abstract

A two-stage surgical procedure involving labioplasty and palatoplasty is a common surgical modality performed in children with cleft lip and palate. Additionally, an alveolar cleft bone graft is performed prior to the eruption of the canine teeth. These three surgeries impose the burden of general anesthesia separately for each procedure, and the formation of scar tissue from the procedure inhibits maxillary growth. We adopted a single-stage surgical procedure to overcome these drawbacks. To date, there have been no reports comparing the treatment outcomes of alveolar morphology and maxillary growth and development in children who underwent single-stage surgery with those who underwent two-stage surgery using plaster casts and cephalograms. Twenty children aged 5–7 years were equally divided into two groups based on whether they had undergone a two- or single-stage procedure. Cephalometric analysis and analysis of dentition models were conducted. The results showed that the single-stage surgery exhibited significant differences in the sella-nasion angle, point A to McNamara line, maxillary length, mandibular body length, and posterior arch width and length compared with the two-stage surgery. Therefore, it was suggested that the single-stage surgery had a favorable effect on maxillary growth compared with the two-stage surgery.

## 1. Introduction

Children with cleft lip and palate commonly undergo a two-stage surgical procedure, with labioplasty at 3 months of age and palatoplasty at 1 year and 6 months of age, both of which are conducted under general anesthesia. Additionally, an alveolar cleft bone graft is required prior to the eruption of the canine teeth. However, these three operations place a great burden on the patient’s entire body for each operation. Moreover, the scar shrinkage caused by each procedure inhibits maxillary growth. To avoid these risks, the Department of Orthodontics at Ou University Dental Hospital and the Department of Plastic and Reconstructive Surgery at Fukushima Medical University Hospital adopted an interdisciplinary approach. After preoperative orthognathic treatment at 5 months of age, single-stage surgery was performed, wherein labioplasty, palatoplasty, and gingivoperioplasty were performed simultaneously [1]. This allows for the complete surgical procedure to be conducted in one session; therefore, treatment is possible with a single exposure to general anesthesia and the minimal risk of complications and formation of scar tissue.

To date, there have been no reports comparing the treatment outcomes of alveolar morphology and maxillary growth and development in children who underwent single-stage surgery with those who underwent two-stage surgery using plaster casts and cephalograms. In this study, we comparatively analyzed the therapeutic effects and usefulness between two-stage surgery and single-stage surgery, wherein children with cleft lip and palate underwent preoperative orthognathic treatment using a nursing-type palatal plate (henceforth, “molding plate” [2]) at the early postnatal period [3,4,5] and subsequently underwent single-stage surgery.

## 2. Materials and Methods

### 2.1. Patients

This study included children aged 5–7 years with unilateral cleft lip and palate who had undergone treatment. Ten children (five boys and five girls; age: 5.8 ± 0.6 years) who underwent single-stage surgery were assigned to the single-stage surgery group. Ten children (six boys and four girls; age: 5.6 ± 0.6 years) who underwent conventional labioplasty and palatoplasty in two surgical stages were assigned to the two-stage surgery group. The study protocol was reviewed and approved by the Ethics Committee of the Faculty of Dentistry, Ohu University (approval number: 305; approval date: 7 September 2020; facility number: 11000803). The parents of the 20 infants provided informed consent.

### 2.2. Surgical Techniques

#### 2.2.1. Single-Stage Surgery

**Figure 1:**
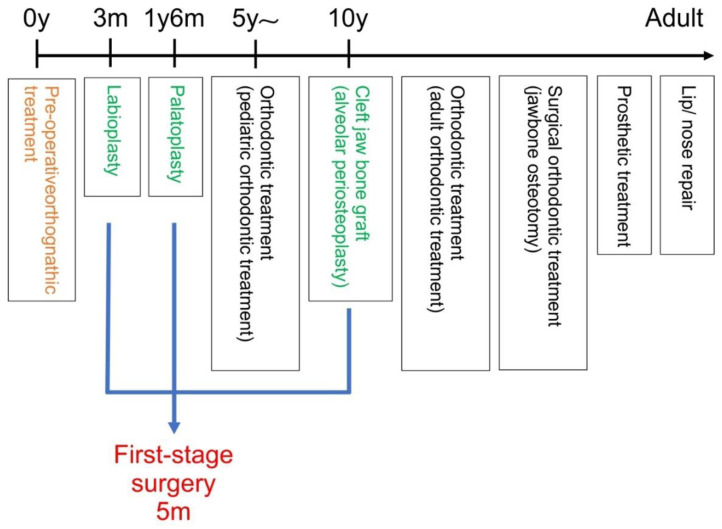
Flowchart of the treatment process with single-stage surgery.

**Figure 2:**
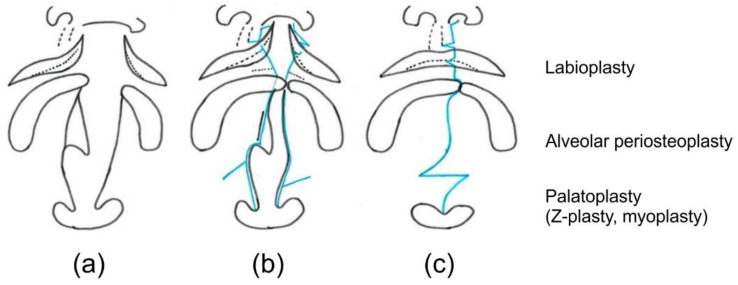
Schematic diagram of the single-stage surgery. (**a**) Unilateral cleft lip and palate, (**b**) incisions, and (**c**) completion of surgery.

Initially, the alveolar cleft width was reduced to ≤2 mm using a molding plate during preoperative orthognathic treatment. Subsequently, labioplasty (modified small triangular flap method), palatoplasty (Furlow method [6]), and alveolar periosteoplasty [7,8] were conducted together in a single surgical session at 5 months of age (Figure 1 and Figure 2).

#### 2.2.2. Two-Stage Surgery

This surgical procedure is the conventional method that is commonly conducted to date, wherein labioplasty is conducted at 3 months of age, and palatoplasty (mainly the push-back method [9,10]) is conducted at 1 year and 6 months of age. Alveolar cleft bone grafting is also conducted around the age of 9 years, when the eruption of the canine teeth occurs, to prepare the space for those teeth.

### 2.3. Preoperative Orthognathic Treatment

**Figure 3:**
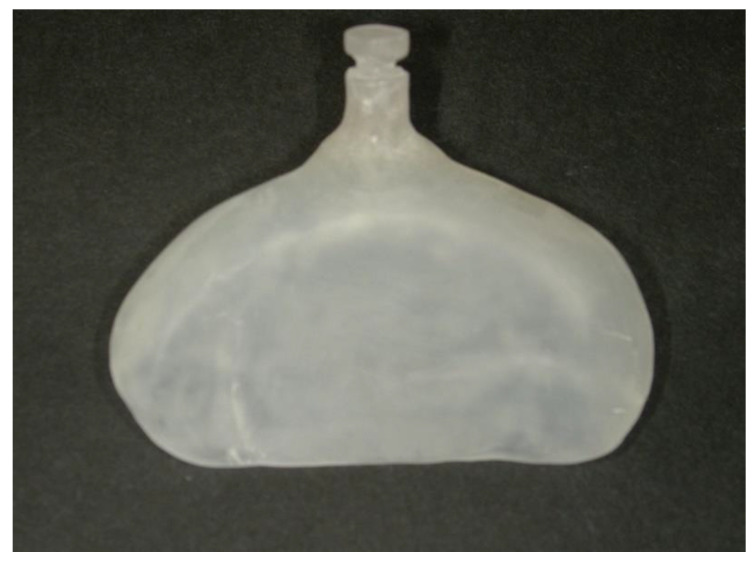
Molding plate used for preoperative orthognathic treatment.

**Figure 4:**
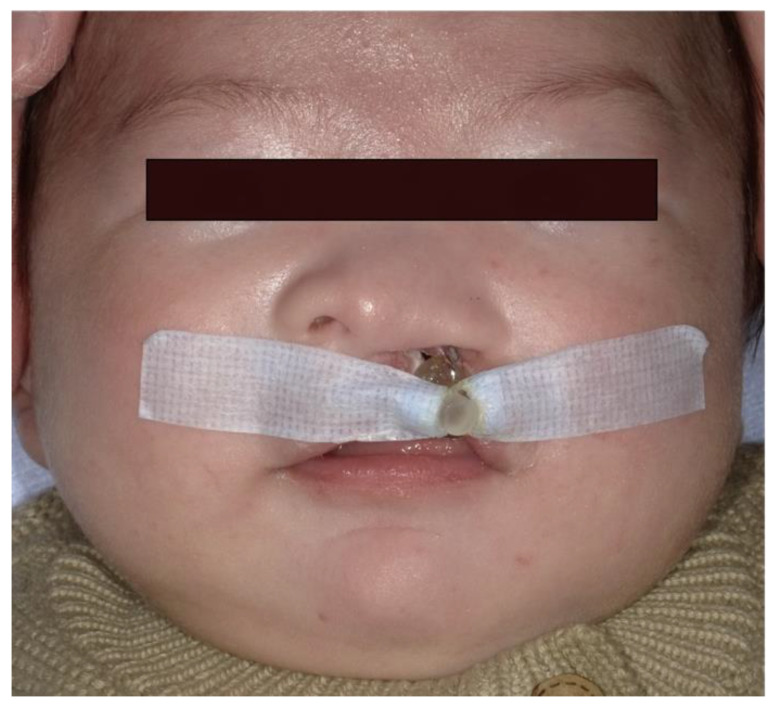
Attachment of the molding plate.

The use of a molding plate (Figure 3) fixed to the patient’s cheeks with orthodontic elastics and medical tape (Figure 4) enables the improvement in feeding problems and narrowing of the cleft width. To prepare the molding plate, an impression was made using a putty-based silicone impression material under airway control in the early postnatal period, and a plaster model was prepared. A wide area from the alveolar cleft to the alveolar crest was then filled flat on the plaster model using molybdenum putty. A molding plate was prepared using instant polymerization resin. The patients started using this appliance on the same day. A new molding plate was prepared approximately every month and was used until immediately prior to the single-stage surgery. The appliances used for preoperative orthognathic treatment and their roles are given below.

#### 2.3.1. Intraoral: Molding Plate

Attaching a molding plate in the oral cavity enables improvements in feeding problems, the induction of jaw development by preventing engagement of the tongue into the jaw cleft, a reduction in the jaw cleft width (by the induction of jaw development), and the flattening of the alveolar ridge morphology [11,12,13,14,15].

#### 2.3.2. Extraoral: Orthodontic Elastics and Medical Tape

Medical tape was used with orthodontic elastics attached to the hook on the anterior part of the palatal plate and fixed to the cheeks. This not only fixes the molding plate in the oral cavity, but also applies tension to the dental arch from the front to the back.

### 2.4. Analyses

The growth and development of the maxillary bone and its alveolar region were investigated using dentition models and lateral cephalometric radiographs and compared between the two groups. Lateral cephalometry involves the measurement of seven parameters of the skeletal system, according to standard methods [16,17,18,19]. The measurement items that were used in the analyses were as follows: the angle between the sella-nasion (SN) plane and straight-line nasion to point A (NA) (SNA angle), the angle between the SN plane and straight-line nasion to point B (NB) (SNB angle), the angle between straight-line NA and straight-line NB (ANB angle), the maxillary length (A’-Ptm’), and the mandibular body length (Go-Me). Furthermore, the relationship between the maxillary and mandibular body lengths was evaluated using the McNamara line, which is a perpendicular line from the nasion (N) of the maxilla to the Frankfurt plane. The distances from the McNamara line to the A point and pogonion (Pog) were measured.

We used a dentition model to measure the dental arch morphology and measured the width and length of the maxillary arch. The measurements were obtained from the anterior and posterior regions of the arch.

#### 2.4.1. Measurement Points in the Lateral Cephalometric Radiographs (Figure 5)

S: midpoint in the urn-shaped shadow of the sella turcica of the sphenoid bone.

N: foremost point of the fronto-nasal suture.

Or: lowest point on the orbital rim.

Po: uppermost point on the upper edge of auditory meatus.

A: deepest point on the maxillary outline between the anterior nasal spine and alveolar crest between the maxillary central incisors.

B: deepest point on the mandibular outline between the alveolar crest of the mandibular central incisors and pogonion.

ANS: apical point of the anterior nasal spine.

PNS: tip of the posterior nasal spine.

Ptm: lowest point of the pterygopalatine fossa.

Go: point where the bisector of the angle formed by the mandibular marginal plane and the mandibular ramus plane intersect the outline of the mandibular angle.

Me: lowest point on the outline of the midline cross-sectional image of the mandibular symphysis.

Pog: anterior-most point on the outline of the midline cross-sectional image of the mandibular symphysis.

Ar: point where the shadow of the margin of the base of the skull intersects the posterior margin of the mandibular ramus.

A’: point where the perpendicular line drawn from A to the NF plane intersects the NF plane.

Ptm’: point where the perpendicular line drawn from Ptm to the NF plane intersects the NF plane.

**Figure 5:**
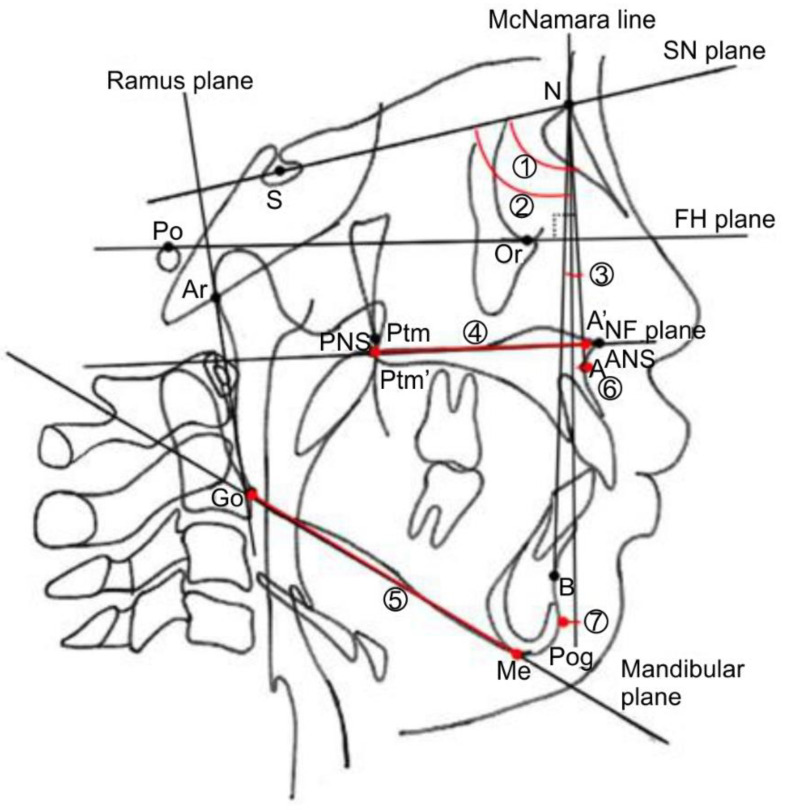
Measurement sites in lateral cephalometric radiograph.

#### 2.4.2. Measurement Planes in Lateral Cephalometric Radiographs (Figure 5)

SN plane: straight line connecting S and N.

FH plane: Frankfurt horizontal plane; straight line connecting OR and Po.

NF plane: straight line connecting ANS and PNS.

Mandibular marginal plane: tangential line drawn from the posterior part of the mandibular margin and the lower edge of the median cross-sectional image of the mandibular symphysis.

Mandibular ramus plane: tangential line drawn from the Ar to the posterior edge of the mandibular ramus.

McNamara line: perpendicular line from the N to FH plane.

#### 2.4.3. Measurement Sites on Lateral Cephalometric Radiographs (Figure 5)

① SNA angle: angle between the SN plane and straight-line NA.
② SNB angle: angle between the SN plane and the straight-line NB.
③ ANB angle: angle between the straight lines AN and NB.
④ A’-Ptm’: maxillary length.
⑤ Go-Me: mandibular body length.
⑥ A to the McNamara line: distance from the McNamara line to point A.
⑦ Pog to McNamara line: distance from the McNamara line to Pog.

#### 2.4.4. Measurement Sites in the Dentition Model (Figure 6)

① Anterior width of the dental arch: distance between the maxillary left and right deciduous canines.
② Posterior width of the dental arch: distance between the maxillary left and right second deciduous molars.
③ Anterior length of the dental arch: distance from the labial surface of the maxillary central incisor to the maxillary left and right deciduous canines.
④ Posterior length of dental arch: distance from the labial surface of the maxillary central incisor to the maxillary left and right second deciduous molars.

**Figure 6:**
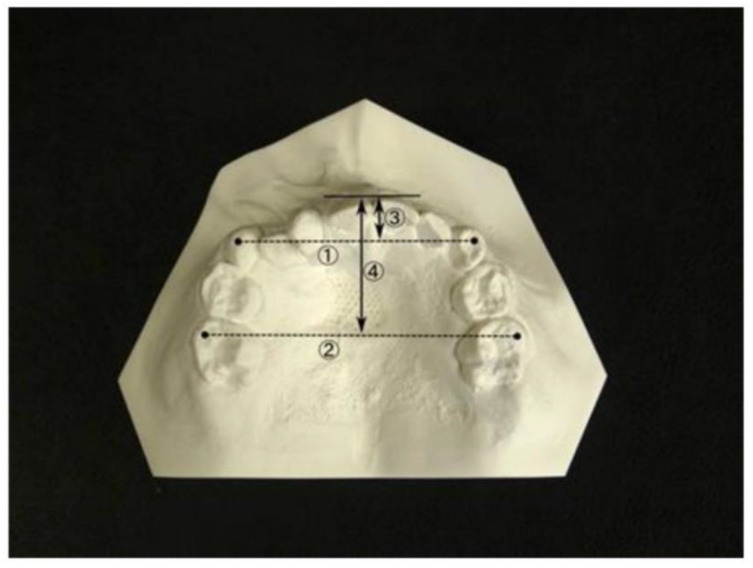
Measurement sites in the dentition model.

### 2.5. Statistical Analysis

Statistical analysis software (SPSS 24.0, IBM Corp., Armonk, NY, USA) was used for the statistical analysis. Comparisons between the single- and two-stage surgery groups were conducted using the Mann–Whitney U test. The level of significance was set at *p* < 0.05.

## 3. Results

The lateral cephalometric radiographs showed significant differences in the following four parameters: SNA, A to McNamara line, A’-Ptm’, and Go-Me. No significant differences were observed between the SNB, ANB, and Pog to McNamara line. The analysis results of the dentition model showed significant differences in the two items of posterior width and length of the dental arch; however, no significant differences were noted in the anterior width and length of the dental arch (Table 1 and Table 2).

## 4. Discussion

In recent years, many postoperative evaluations have been conducted among Japanese children with unilateral cleft lip and palate using the 5-year-olds’ index, and its usefulness has been established [20]. Therefore, this study examined children aged 5–7 years with unilateral cleft lip and palate. Regarding sex, there were five boys and five girls in the single-stage surgery group and six boys and four girls in the two-stage surgery group; the sex ratio between the two groups was almost identical. Therefore, the results were not influenced by sex differences.

### 4.1. Preoperative Orthognathic Treatment

Preoperative orthognathic treatment using a molding plate not only improves feeding problems, but also induces jaw growth by preventing the tongue from intruding into the jaw cleft. Furthermore, Torikai et al. [1] reported that the cleft width could be reduced to ≤2 mm even in patients with a large cleft width, making single-stage surgery possible. It is also believed that scar tissue formation can be minimized by narrowing the jaw cleft width using a molding plate. Furthermore, the molding plate could be flattened by filling a wide area from the alveolar cleft to the alveolar crest and hard palate with molybdenum putty on the plaster model while making the impression. Therefore, clefts can be narrowed even if they are present, and if palatoplasty causes scar shrinkage, growth suppression of the posterior part of the dental arch can be minimized.

### 4.2. Single-Stage Surgery

Single-stage surgery enables the three surgeries of labioplasty, palatoplasty, and alveolar periosteoplasty to be conducted together; this not only reduces the physical burden imposed on the patient and family, but also reduces the risks associated with multiple exposures to general anesthesia. Furthermore, the Furlow method is the first-line treatment in single-stage palatoplasty. The push-back method, which is conducted at many facilities, involves the formation of a mucoperiosteal flap on the hard palate and lifting it, after which it is moved backward, thereby exposing part of the maxilla in the oral cavity. This causes strong scar shrinkage and hinders the growth of the maxilla, which causes reverse occlusion [21]. In contrast, the Furlow method involves Z-plasty on the soft palate and aims to lengthen the soft palate and muscle formation simultaneously, avoiding the exposure of the maxillary bone in the oral cavity. Therefore, its effect on jaw development can be minimized. However, when the width of the cleft palate is large, a relaxing incision needs to be added to the Furlow method. Therefore, preoperative orthognathic treatment was performed as a compensatory measure. Preoperative orthognathic treatment can significantly narrow the cleft width; therefore, conducting a single-stage surgery that minimizes the impact on jaw development is possible.

### 4.3. Efficacy Compared with Two-Stage Surgery Based on Measurement Results

Conducting surgery at an early stage may alter the impact on growth and development. Furthermore, the push-back method was used in the two-stage surgery; hence, there was a difference between the surgical procedures depending on the presence or absence of scar tissue formation in the hard palate. This may have resulted in significant differences in the measurements in the posterior part of the dental arch. It has been reported that reducing the maxillary cleft width to ≤2 mm by preoperative orthognathic treatment eliminates the need for a relaxing incision due to the Furlow method during single-stage surgery [1]. As it prevents the maxilla from growing forward and laterally owing to the formation of scar tissue, this may reduce the risk of future reverse occlusion.

### 4.4. Future Directions

Single-stage surgery suppresses the appearance of scar tissue in patients with cleft palate, and it is an advantageous surgical procedure for obtaining normal occlusion with less systemic burden on the patient. However, few facilities have the infrastructure to provide single-stage surgery. In the future, as single-stage surgery becomes accepted not only nationwide, but also worldwide, stable occlusal recovery in patients with cleft palate can be expected. In this study, we consider that long-term observation is necessary as the children are still growing and developing. Therefore, this study is preliminary and we will continue to study the progress.

## 5. Conclusions

In this study, single-stage surgery, which is a new modality for treating cleft lip and palate, reduced growth inhibition in the maxilla in children with unilateral cleft lip and palate, suggesting the effectiveness of single-stage surgery over the conventional two-stage surgery.

## Figures and Tables

**Table 1:** Cephalometric analysis results.

Measurement Item	Single-Stage Surgery (*n* = 10)		Two-Stage Surgery (*n* = 10)		*p*-Value
	Mean	SD	Mean	SD	
SNA (°)	78.92	1.61	76.78	2.70	0.035 *
SNB (°)	77.45	3.49	77.43	3.39	n.s.
ANB (°)	1.49	1.95	4.9	1.95	n.s.
A’–Ptm’ (mm)	43.41	2.24	43.25	1.4	0.004 *
Go–Me (mm)	53.47	3.72	61.19	4.08	0.001 *
A to McNamara line (mm)	−3.71	1.53	−5.55	2.20	0.043 *
Pog to McNamara line (mm)	−10.92	5.67	−9.41	5.59	n.s.

SD, standard deviation; n.s., not significant; SNA, angle between the sella-nasion (SN) plane and straight-line nasion to point A; SNB, angle between the SN plane and straight-line nasion to point B (NB); ANB, angle between straight-line NA and straight-line NB; A’–Ptm’, maxillary length; Go–Me, mandibular body length. Analyses were performed using the Mann–Whitney U test. * *p* < 0.05.

**Table 2:** Analysis results of the dentition model.

Measurement Item	Single-Stage Surgery (*n* = 10)		Two-Stage Surgery (*n* = 10)		*p*-Value
	Mean	SD	Mean	SD	
Anterior width of dental arch (mm)	28.06	1.57	25.53	4.04	n.s.
Posterior width of dental arch (mm)	48.31	2.46	42.80	1.19	0.001 *
Anterior length of dental arch (mm)	4.48	0.84	4.50	1.21	n.s.
Posterior length of dental arch (mm)	19.31	1.58	17.40	1.49	0.029 *

SD, standard deviation; n.s.: not significant. Analyses were performed using the Mann–Whitney U test. * *p* < 0.05.

## Data Availability

Not applicable.
